# Improving Behavioral Support for Smoking Cessation in Pregnancy: What Are the Barriers to Stopping and Which Behavior Change Techniques Can Influence Them? Application of Theoretical Domains Framework

**DOI:** 10.3390/ijerph15020359

**Published:** 2018-02-17

**Authors:** Katarzyna A Campbell, Libby Fergie, Tom Coleman-Haynes, Sue Cooper, Fabiana Lorencatto, Michael Ussher, Jane Dyas, Tim Coleman

**Affiliations:** 1 Division of Primary Care, University of Nottingham, Room 1406, Tower Building, University Park, Nottingham NG7 2RD, UK; msxebf@exmail.nottingham.ac.uk (L.F.); msatc5@exmail.nottingham.ac.uk (T.C.-H.); sue.cooper@nottingham.ac.uk (S.C.); manerba.it@hotmail.co.uk (J.D.); tim.coleman@nottingham.ac.uk (T.C.); 2 Centre for Behaviour Change, University College London, London WC1E 7HB, UK; f.lorencatto@ucl.ac.uk; 3 Division of Population Health Sciences and Education, St George’s University of London, London SW17 ORE, UK; mussher@sgul.ac.uk; 4 Institute of Social Marketing, University of Stirling, Stirling, FK9 4LA Scotland, UK

**Keywords:** smoking cessation, pregnancy, behavior change techniques, intervention development, Theoretical Domains Framework

## Abstract

Behavioral support interventions are used to help pregnant smokers stop; however, of those tested, few are proven effective. Systematic research developing effective pregnancy-specific behavior change techniques (BCTs) is ongoing. This paper reports contributory work identifying potentially-effective BCTs relative to known important barriers and facilitators (B&Fs) to smoking cessation in pregnancy; to detect priority areas for BCTs development. A Nominal Group Technique with cessation experts (*n* = 12) elicited an expert consensus on B&Fs most influencing women’s smoking cessation and those most modifiable through behavioral support. Effective cessation interventions in randomized trials from a recent Cochrane review were coded into component BCTs using existing taxonomies. B&Fs were categorized using Theoretical Domains Framework (TDF) domains. Matrices, mapping BCT taxonomies against TDF domains, were consulted to investigate the extent to which BCTs in existing interventions target key B&Fs. Experts ranked ‘smoking a social norm’ and ‘quitting not a priority’ as most important barriers and ‘desire to protect baby’ an important facilitator to quitting. From 14 trials, 23 potentially-effective BCTs were identified (e.g., ‘information about consequences). Most B&Fs fell into ‘Social Influences’, ‘Knowledge’, ‘Emotions’ and ‘Intentions’ TDF domains; few potentially-effective BCTs mapped onto every TDF domain. B&Fs identified by experts as important to cessation, are not sufficiently targeted by BCT’s currently within interventions for smoking cessation in pregnancy.

## 1. Introduction

Smoking in pregnancy is detrimental to both mothers’ and babies’ health [1,2,3]. It is a significant public health concern in developed countries—for example, around 10% of pregnant women are reported to smoke in the UK [4] and Canada [5], and 8.4% in the U.S. [6]. Younger women, those in routine or manual occupations and those who never worked, as well as women who live with at least one smoker are more likely to smoke throughout their pregnancy [7]. Women who are married/living with partner and have support from their partner or other family member are less likely to smoke in pregnancy [8] and those who do smoke, are more likely to quit in pregnancy [9]. Pregnancy is a special time in women’s lives during which many are motivated to quit and try stopping [10]. Reducing smoking in pregnancy is a target of health organizations worldwide [11,12]; behavioral support interventions can help encourage smoking cessation and should be offered to pregnant women to improve their chances of permanently stopping smoking [13], however more research is necessary to help establish which aspects of the interventions are effective.

Development of behavioral interventions should be guided by appropriate behavior change theories [14] and the efficacy of behavioral smoking cessation interventions will likely be maximized if these address determinants of cessation and successfully target barriers against and facilitators for (B&Fs) stopping smoking in pregnancy [15]. Additionally, interventions should be comprised of relevant and effective behavior change techniques (BCTs), which help overcome specific barriers against or amplify particular facilitators towards stopping smoking in pregnancy [15]. BCTs have been defined as interventions’ ‘active ingredients’ which are ‘observable, replicable and irreducible components of an intervention designed to alter or redirect causal processes that regulate behavior’ (e.g., goal setting, problem solving, action planning) [16].

For stopping smoking in pregnancy, a systematic review of 38 qualitative studies collated B&Fs to cessation reported by pregnant smokers [17]. These B&Fs were classified into four major domains relating to women’s psychological wellbeing, their relationships with others, understanding the risks of smoking in pregnancy, and the changing connection with the baby throughout the pregnancy. Another review [18] identified component BCTs in smoking cessation interventions for pregnant women, for which there was randomized controlled trial (RCT) level evidence of effectiveness. A systematic method of identifying component BCTs within behavior change interventions are BCT taxonomies, which describe individual BCTs consistently and organize them hierarchically into broader categories [16,19]. The authors of the review [18] employed a smoking-specific taxonomy comprising 43 BCTs [19]; they found that only 11 of these BCTs were deployed within effective interventions, including ‘information about consequences of smoking/cessation’, ‘goal setting’ and ‘relapse prevention’. Furthermore, only 15% of treatment manuals used by UK Stop Smoking Services advocated smoking cessation advisors use all 11 of the effective BCTs [18]. The extent to which BCTs used in existing effective interventions target key B&Fs to smoking cessation in pregnancy is currently unclear.

The Theoretical Domains Framework (TDF) is an integrative theoretical model that synthesizes main behavior change constructs across key theories, into 14 domains, such as ‘Knowledge’ or ‘Goals’ [20]. It has been used to inform development of behavior change interventions [21,22], including smoking cessation [23]. The application of the TDF can help highlight in which domains the key B&Fs to smoking cessation in pregnancy lie and help identify BCTs that might effectively target these. 

As part of a larger project, which aims to develop an improved behavioral smoking cessation intervention for pregnant women, this paper describes the first parts of the process and relates these findings. In this initial work, we aimed to derive an expert consensus on the B&Fs that most influence women’s smoking cessation and are most modifiable through behavioral support; to describe any BCTs used in recent behavioral cessation interventions for pregnant women with randomized controlled trial-evidence of effectiveness [18]; and, in the context of the Theoretical Domains Framework (TDF) [20], to match all these potentially-effective BCTs to B&Fs. Thus we aimed to identify all tested BCTs with evidence of effectiveness and, using an appropriate theoretical framework, to relate these to important but modifiable B&Fs to help priorities future BCT development such that this might be expected to maximally increase the effectiveness of behavioral interventions for stopping smoking in pregnancy.

## 2. Materials and Methods

Ethical approval was obtained from East of Scotland Research Ethics Service REC 1, REC reference number 16/ES/0125. 

### 2.1. Step One: Identification of Key Barriers and Facilitators to Smoking Cessation in Pregnancy

We used Nominal Group Technique to generate experts’ consensus about which B&Fs are perceived most likely to influence smoking cessation in pregnancy [24]. Nominal Group Technique uses facilitated, structured face-to-face group interactions between experts to generate new ideas and usually starts with independent generation of ideas, which are shared with the group and discussed within the group until a final, ranked list of ideas is agreed [24,25].

#### Procedure

The recommended number of participants in Nominal Group Technique meetings is 9 to 12 [25]. We aimed to recruit experts within the UK who had practical and/or theoretical knowledge of providing smoking cessation support in pregnancy, so we purposefully selected researchers, public health specialists and cessation advisors working within the field of smoking in pregnancy, but aiming for a group comprised predominantly of cessation advisors who specialized in supporting pregnant women. As the provision of smoking cessation support in pregnancy differs across the country, we invited experts from different areas of the UK to participate, by sending a study description, participant information sheet and a consent form. The meeting was held on 30 January 2017; before giving consent, participants were asked to treat discussions as confidential and informed that these would be audiotaped and that anonymized quotes might appear in publications.

The meeting was facilitated by JD, supported by KAC and LF, and observed by SC. At this meeting, participants were first asked to list five barriers and facilitators (B&Fs) which they perceived as having the most influence on pregnant women’s chances of stopping smoking and which they also believed could be addressed by cessation advisors in behavioral support sessions. Next, participants were given a list of 25 B&Fs to cessation taken from a systematic review of qualitative studies investigating pregnant women’s views [17]. Participants were asked to compare this to their own list and to highlight any ‘new’ B&Fs that were not on the systematic review list; the group then discussed each ‘new’ B&F to determine whether or not this was indeed original and if it could be addressed in support sessions. These ‘original and new’ B&Fs were added to the review B&Fs to form a combined list of B&Fs which might influence pregnant women’s smoking behavior and which were also judged amenable to manipulation in support sessions; we refer to these as ‘*individual*’ B&Fs and these were used in further work. Any potentially-important B&Fs that were identified, but which could predominantly be addressed by organizational-level actions rather than by advisors in behavioral support sessions were termed ‘*environmental*’ B&Fs and were not discussed further.

From the combined list, each participant ranked their top 10 B&Fs in terms of influence on pregnant women’s chances of stopping smoking (1st = greatest influence) and we then allocated scores (1–10) to individuals’ rankings whereby the highest ranked B&Fs were given the highest score (i.e., 1st ranked scored 10, 2nd ranked scored 9 etc.). We added all individual participants’ scores for each B&F; those with highest total-score were considered to be the most influential. We then re-presented the participants with a list of the 10 B&Fs with the highest-total score to represent group consensus on those B&Fs perceived most influential on women’s smoking cessation. Participants were then asked to rank these 10 ‘most influential’ B&Fs in terms of how easy/difficult they might be to address in behavioral support sessions (1st ranked = most difficult to address; scored 1, 2nd ranked scored 2 etc). Again, we added the scores for each of these 10 B&Fs; those with highest total-score were considered the easiest to address.

### 2.2. Step Two: Identification of Behaviour Change Techniques Used in Effective Interventions

Using methodology described by Michie et al. [19] and replicated by Lorencatto et al. [18] we identified BCTs used in smoking cessation interventions in pregnancy for which there was randomized control trial-level (RCT) evidence of effectiveness. From the most recent Cochrane review of behavioral support interventions for smoking cessation in pregnancy [13], we selected RCTs in which a behavioral support intervention was compared with a relevant control (often usual care). We considered that interventions showed evidence of effectiveness when, following intervention, there were statistically significant differences in cessation rates between trial groups at any point during pregnancy. Statistical significance was defined as an Odds Ratio (OR) >1.0, with 95% confidence intervals where the lower limit was >1.0 or a *p* value of <0.05.

From the trials with effective interventions, we specified interventions’ content in terms of component BCTs using the Behavior Change Techniques Taxonomy version 1 (BCTTv1) as a coding framework [16], supplemented by a taxonomy describing smoking cessation-specific BCTs and competencies (i.e., techniques necessary for effective delivery of behavior change techniques, but not able to change behavior in their own right) [19]. Four authors (LF, KAC, TCH and MU) mapped the 53 smoking-specific BCTs/competencies [19] onto the BCTTv1 [16]; 33 smoking-specific BCTs were mapped on to BCTs in BCTTv1, for example ‘advise on/facilitate use of social support’ was mapped onto ‘social support’. The remaining 20 smoking-specific items, which were all considered to be competencies rather than BCTs, related to the general aspects of interactions and did not correspond to any items within BCTTv1. Consequently, the taxonomy used to code the contents of interventions comprised 113 items, 93 of which were BCTTv1 BCTs, and 20 were smoking-specific competencies (see List A1, Appendix).

#### Data Analysis—Behavior Change Technique Coding

Researchers (KAC and TCH) were trained in BCT coding (see http://www.bct-taxonomy.com) before analyzing the text describing each effective intervention in trial papers and coding for BCTs/competencies present using the taxonomy above. We report the taxonomy categories of BCTs/competencies, the numbers of BCTs and competencies per effective intervention and number of interventions in which each BCT was included. We compared initial agreement between coders, using Cohen’s kappa [26]. Where coders disagreed, consensus was sought via discussion or, if necessary by consulting a third researcher (FL). We considered a BCT potentially-effective if it had been used in two or more effective interventions as defined above [18].

### 2.3. Step Three: Theoretical Domains Framework Mapping

We employed the Theoretical Domains Framework (TDF) as our analytical framework [27] to match potentially-effective BCTs to key B&Fs to smoking cessation in pregnancy. LF and KAC independently mapped the list of combined B&Fs (see Section 2.1) onto the 14 TDF domains [28]. For example, the B&F ‘women’s lack of understanding of issues of safety around using NRT in pregnancy’ was coded into the ‘Knowledge’ domain. They compared mappings and if a barrier or facilitator was mapped onto more than one domain, discussed which domain was the most appropriate, resolving discrepancies until consensus was reached and each B&F was mapped onto one domain that was thought to best reflect its nature. Interrater reliability of the initial coding was calculated using Cohen’s kappa [26].

Past studies have generated expert consensus to develop mapping matrices of TDF domains and BCTs with potential to influence each domain [28,29]. For example, BCT ‘information about health consequences’ was linked to the ‘Knowledge’ domain. We used the matrix linking BCTTv1 with TDF domains [28] to code the BCTs with RCT evidence of effectiveness (see Section 2.2) onto the TDF domains. LF, KAC and TCH also coded onto the domains those BCTs for which there was no past consensus regarding which domain they can be linked to. The competencies were not mapped onto TDF domains, as while they are necessary for intervention delivery, they do not directly influence behavior. 

The mapping of the B&Fs and BCTs with RCT evidence of effectiveness to relevant TDF domains, was conducted in order to help priorities areas for which future BCTs should be developed to maximally increase the effectiveness of behavioral smoking cessation in pregnancy interventions.

## 3. Results

### 3.1. Step One: Identification of Key Barriers and Facilitators to Smoking Cessation in Pregnancy

We invited 28 experts; 19 expressed interest and 12 attended the meeting. Participants came from the East Midlands, West Midlands, North West and East England, and from Scotland; eight were smoking cessation advisors who provided support to pregnant smokers, three were researchers/academics in the field of smoking cessation in pregnancy and one was a public health specialist with experience and knowledge of smoking cessation issues at policy level. They had a mean (SD) 9.96 (5.42) years of experience in a relevant role.

Twenty three potentially ‘new’ B&Fs, which were perceived influential for pregnant women’s chances of stopping smoking and addressable in support sessions, were identified initially; after discussion, the group decided that 14 of these were original (Table 1). Ten were ‘*individual*’ B&Fs considered addressable in support sessions; these related to women’s knowledge or use of NRT, motivation/self-belief in ability to quit, and communication with health professionals. The remaining four B&Fs were deemed ‘*environmental*’ and likely to require organization-level action to be addressed (e.g., changes to referral systems). Participants’ rankings of the B&Fs are shown in Table 2; the 10 perceived as most influential for pregnant women’s chances of stopping smoking are presented and the ease of addressing these B&Fs within behavioral support sessions is also shown. Ranked B&Fs were selected from a combined list of 35 B&Fs, the 10 ‘individual and new’ B&Fs added to the previously-identified 25 B&Fs [17].

### 3.2. Step Two: Identification of Behaviour Change Techniques Used in Effective Interventions

The Cochrane review [13], which we used as a starting point to identify trials of effective interventions, included 102 trials; 70 were RCTs or cluster RCTs of behavioral support interventions for smoking cessation during pregnancy and of these, 56 tested interventions which were ineffective as judged by our inclusion criteria, leaving for further scrutiny 14 trials in which there was a statistically significant improvement in quit rates in pregnancy in experimental group vs. the control [30,31,32,33,34,35,36,37,38,39,40,41,42,43]. These 14 trials comprised 18 study arms in which interventions were tested; two studies had two intervention arms [36], and one had three [37], however only one arm in each of the trials showed evidence of effectiveness and so we coded BCTs within 14 intervention arms. These trials were mainly conducted in USA [30,31,32,33,37,42,43] and Europe [34,35,36,38,39,40]. Nine interventions were delivered face to face or over the telephone by antenatal staff or other trained professionals [31,32,33,34,38,39,40,41,42]; the majority of these also offered written materials and two included computer or video delivered materials. The remaining interventions were delivered predominantly as printed materials [30,35,36], videotape [43] and via a computer [37]. Only two studies [37,39] were published since 2009, which was the cut-off point for the previous review [18,44].

#### 3.2.1. Interrater Reliability

For eight studies, coders’ independent ratings reached perfect or almost perfect agreement (Cohen’s kappa >0.80); for five there was substantial agreement (Cohen’s kappa >0.60) and for one study agreement was moderate (Cohen’s kappa = 0.55) [26].

#### 3.2.2. Behavior Change Techniques Content of the Interventions

The 14 interventions included a mean (SD) of 9.5 (3.03) BCTs/competencies. Twenty three (20%) of the 113 BCTs/competencies described in the combined taxonomy were identified as components of two or more interventions found to be effective in RCTs (16 BCTs and 8 competencies; 8 of these BCTs and 5 competencies have not been previously identified in effective interventions for smoking cessation in pregnancy [18]; see Table 3). One BCT, ‘information about health consequences’ of smoking/smoking cessation, was present in all 14 interventions. ‘Biofeedback’, using exhaled carbon monoxide levels or cotinine levels, was utilized in 86% of interventions. Overall, BCTs relating to ‘goals and planning’ were identified in 79% interventions; 64% of interventions used a credible expert to convey information (e.g., midwife, obstetrician); and 50% used ‘social support’—either in the form of offering additional cessation support or, less often, including a ‘buddy’: a close one who would support cessation attempt. Most (93%) interventions incorporated competencies relating to ‘general aspects of communication focusing on gathering information’ about smoking behaviors or motivation to quit. Approximately one third of interventions described competencies aimed to tailor interactions to client’s needs. The frequencies of BCTs/competencies identified in two or more effective trials are presented in Table 3.

### 3.3. Step Three: Theoretical Domains Framework Mapping

Table 4 shows how the 35 B&Fs mapped on to the TDF and highlights the 10 B&Fs, which were identified as having greatest influence on women’s smoking, in the expert group consensus. B&Fs mapped to eight (of 14) TDF domains: ‘Social influences’, ‘Knowledge’, ‘Emotions’, ‘Intentions’, ‘Beliefs about Capabilities’, ‘Environmental Context’, ‘Optimism’ and ‘Social Role’. Within these eight domains there was a mean (SD) of 4.38 (3.81) B&Fs mapped to each domain. The interrater reliability was perfect (kappa = 1) for 27 B&Fs, substantial (kappa > 0.6) for seven, and moderate (kappa = 0.44) for one.

Table 4 also shows that at least one potentially-effective BCT mapped onto the same seven TDF domains as the B&Fs; no BCT mapped onto the ‘Social Role’ domain. Within the eight domains there was a mean (SD) of 1.5 (1.06) BCTs mapped. 

We mapped many B&Fs to some of the domains, but then identified few BCTs that could potentially address these B&Fs. For example, 12 B&Fs, predominantly related to social norms and family influences, mapped within the ‘Social Influences’ domain and two of these were identified by group consensus as potentially influential for women’s chances of stopping smoking, but only three potentially effective BCTs mapped to this domain. 

## 4. Discussion

This is a novel, in-depth study which utilized a comprehensive theoretical framework and triangulated data from multiple sources, to explore gaps in the BCT provision in existing effective smoking cessation interventions for pregnant women with the view to guide development of new ones.

To our knowledge, this is the first attempt to rank B&Fs for stopping smoking in pregnancy in terms of their influence and difficulty to address in behavioral support. We found that the most influential barriers to cessation were ‘smoking being a social norm’ and that ‘smoking cessation does not appear to be a priority in women’s lives’; at the same time, these were two of the most difficult to address in support sessions. ‘Women’s desire to protect their child’ was an important facilitator, and experts considered that this could more easily be built upon in behavioral support sessions.

We identified 14 trials which effectively helped women to stop smoking in pregnancy, only two since the previous 2009 Cochrane Review [44]. We identified eight new potentially-effective BCTs and five competencies, such as employing credible experts to deliver interventions, identifying pros and cons to smoking/cessation, and tailoring intervention contents to women’s circumstances, in addition to all BCTs and competencies previously identified [18], resulting in a total of 16 BCTs and 8 competencies used across interventions.

B&Fs to cessation in pregnancy mapped onto eight out of 14 TDF domains, and at least one BCT mapped onto seven of those; however, overall, few BCTs mapped onto each domain. ‘Social Influences’, ‘Intentions’, ‘Environmental Context’, ‘Knowledge’, ‘Beliefs about Capabilities’ and ‘Emotions’ are the domains to which many key B&Fs map but to which we matched few potentially-effective BCTs. Development and testing of BCTs to address these domains could accelerate the production of effective cessation interventions. 

It is important to acknowledge the limitations as well as strengths of this study. A small, purposefully-selected sample, with only one group of participants poses a limitation to generalizability of findings from the expert group; however the group was comprised of various experts in the field with different experiences and expertise, offering a multitude of perspectives. In the current work we did not include service users, however findings from a recent comprehensive systematic review were used to account for women’s views [17]. We used a careful and systematic process to generate a comprehensive list of B&Fs, using data from a systematic review [17] and a nominal group technique, which could be used as basis for further research. Notably, a large proportion of B&Fs identified by the experts in this study as most important, were selected from the B&Fs derived from the expert panel discussion, as compared to B&Fs derived from the systematic review. This might be a significant source of bias; we have since conducted an online questionnaire study based on Delphi consensus building technique [45], to allow a larger group of experts previously not involved in creating the B&Fs list, to provide their views on the importance and amenability to manipulation of the B&Fs (unpublished data). 

The way effective BCTs were identified also has limitations. To fully understand why an intervention was effective, key elements, such as characteristics/skills of the person delivering it, intensity, fidelity of delivery etc. should be considered, as well as the intervention contents [46]. Available descriptions of these factors, however, were often limited, making it difficult to assess how BCTs might be used in practice. Analysis of the ratio of BCTs in effective vs. ineffective trials has been found in the past to provide greater understanding of BCT effectiveness [47]. Using the ratio would have been likely to reduce the list of potentially-effective BCTs identified in the current work; however, as this current study is just our first step in identifying which BCTs could potentially be used when designing future interventions, the method we used allowed us to generate a larger pool of potential BCTs, which could be further developed and tailored for treating pregnant clients. Furthermore, trialed interventions are likely to contain more BCTs than described in published papers [48], but those described are likely to be the main or key BCTs. Finally, our review may not have identified some important BCTs which are in routine clinical use, but haven’t been tested in trials; however, as we intend to use this work for developing interventions that are effective, first looking for BCTs with some evidence for effectiveness is appropriate.

One strength of this study is the systematic approach to identifying potentially-effective BCTs, by using a recent systematic review [13] to identify effective trials of appropriate interventions. We also utilized the recently-developed BCTTv1 [16] together with smoking specific taxonomy [49]. Use of the BCTTv1 ensured that BCT descriptions would be comparable with those made in studies describing BCTs used in interventions to modify other unhealthy behaviors, and use of the smoking-specific taxonomy allowed greater understanding of the techniques used in smoking cessation. 

Application of the TDF [27] is also a strength of this study, as this theoretical framework integrates constructs across several theories. It allowed us to match the potentially-effective BCTs to the key B&Fs, thus identifying relevant life domains which are insufficiently targeted in existing behavioral support and helping to prioritize development of future interventions [20,50]. 

Our findings suggest that for pregnant women, the influence of their social networks is a key domain in which the majority of B&Fs to cessation lie. This is consistent with past research; having a smoking partner and being exposed to second hand smoke are two of the key predictors of smoking in pregnancy [9]. Inadequate support from partners and family members [9] and living within social networks of smokers are also barriers to cessation in pregnancy [51]. In the current study, the fact that smoking is ingrained in women’s complex lives, including their culture and social networks, was considered by the expert group participants to have the highest impact on women’s smoking behaviors, while being the most difficult to address in behavioral support; this closely echoes findings from past studies [52,53], suggesting that these issues should be considered a priority when designing pregnancy specific cessation interventions. Women’s complex lives, and the magnitude of influence that their social networks have on their smoking, requires complex action [51]; our findings suggest, however, that currently there are few potentially-effective BCTs addressing this issue. ‘Social support’ has been identified in half of the interventions in this study, mainly involving support from cessation specialists or a cessation ‘buddy’. This may not be sufficient, and a holistic approach incorporating a number of tailored techniques that could help engage partners and families in cessation process should be considered. In a recent study, expert consensus was reached regarding BCTs that could potentially address B&Fs in all TDF domains, across behaviors [28]. In addition to BCTs identified in our study, Cane and colleagues [28] suggest eight other techniques such as ‘modelling of desired behavior’, which also proved to be effective in a pilot trial with pregnant women [43], ‘social reward’, ‘restructuring of social environment’ and ‘identification of self as a role model’ to address B&Fs relating to social influences; these BCTs could potentially be tailored and developed to specifically address smoking in pregnancy. 

This study also highlights the role of support from health professionals in helping women to quit in pregnancy; for example, clear and consistent communication about smoking and smoking cessation from health professionals, and relationship built on trust and mutual respect were also identified by the expert group participants as important B&Fs to cessation. Using ‘credible experts’, usually midwives, to deliver support was commonly observed across the effective interventions assessed in this study. Midwives are perceived as a credible source of information by women [54], however, they frequently feel underprepared to deliver cessation support [55,56]. Offering comprehensive training to all antenatal care staff could better prepare them to deliver cessation interventions more consistently, capitalizing on the trust women have in them.

Some key barriers relating to the use of Nicotine Replacement Therapy (NRT) mapped onto the ‘Knowledge’, ‘Environmental Context’ and ‘Optimism’ domains. For example, inaccurate assessment of nicotine dependence by health professionals was perceived by the expert group participants to be a potential explanation for inadequate NRT provision, and so hindering quit attempts. While nearly all interventions scrutinized in this study assessed smoking behaviors and motivation to quit, only three used tools to assess nicotine dependence; in the future, accurate assessment could ensure appropriate dose of NRT is given, resulting in fewer cravings. Poor understanding of use and safety of NRT was also highlighted by experts in this study as a barrier to cessation. Past studies found that women are often unaware NRT is permissible in pregnancy, and have insufficient knowledge of NRT risks compared to risks of smoking [57,58,59], which can lead to poor adherence and hinder quit attempts [60]. Nicotine metabolism appears to be faster during pregnancy, increasing women’s need for sufficient doses and consistent use of NRT to aid cessation [61]. While all effective interventions in this study provided information about risks of smoking, more could be done to provide consistent, evidence based advice on NRT use and safety in pregnancy [62]. 

Several barriers to cessation relating to women’s feelings that smoking can help alleviate stress and improve mental well-being mapped onto the ‘Emotions’ domain; one potentially-effective BCT that was found to be appropriate to address B&Fs in the ‘Emotions’ domain was described in the trialed interventions as ‘reducing negative emotions’. Past research, however, indicates that the opposite is true, i.e., that smoking cessation can help reduce anxiety, depression and stress while improving positive affect [63]. Women also see smoking as a way of coping with stress—this coping strategy was associated with greater chance of relapse [64], therefore using BCTs that could help women find alternative ways of dealing with stress could help them stay smoke-free. Providing correct and appropriately pitched ‘information about emotional consequences’ of smoking and quitting, or ‘prompting monitoring of emotional consequences’ of smoking/quitting (e.g., recording how emotions change throughout the day when smoking/staying quit) were considered appropriate by experts in past research to address barriers in this domain [28] and could potentially help pregnant women understand the relationship between smoking and stress.

We also found women’s intentions and self-belief were believed to be important B&Fs to cessation. For example, women’s intention to protect their baby from harms of smoking was identified as an important facilitator, which could be relatively easily addressed in behavioral support; however, smoking is also considered by the women as a low priority in their complex lives. Other authors also found baby’s health coupled with strong internal belief system to be a strong motivator to quit and avoid relapse [9,64]. A number of BCTs relating to goal setting and action planning, which could potentially help strengthen motivation to quit, were identified across trials; however, setting a goal (e.g., quit date) does not always lead to quitting [65], and more work should be invested in developing BCTs to help bridge this gap. ‘Review of goals’, ‘action planning’ and ‘commitment’ were considered to be appropriate to address goals and intentions [28], and could potentially be tailored to the needs of pregnant women.

Finally, three BCTs found in effective trials did not map onto any of the domains in which key B&Fs to smoking cessation lie; these were ‘graded tasks’, ‘pros and cons’ and ‘reward based on outcome of behavior’. ‘Graded tasks’ mainly referred to gradually cutting-down cigarettes. One study suggest that cutting-down does not result in reduction of toxins in the urine and blood stream [66]; furthermore cutting-down is not recommended in pregnancy by National Institute of Health and Clinical Excellence, and health professionals are encouraged to give a clear and consistent advice to stop abruptly, as giving women mixed messages reduces their chance to quit [67]. Financial rewards have been found to significantly increase quit rates in pregnancy [13], but it remains unclear if they are publically and ethically acceptable [68,69]. Nevertheless, the use of financial incentives might be considered with women who failed to quit using other methods; non-financial and self-reward could be an acceptable alternative to boost motivation to quit [38,41]. Asking women to list the ‘pros and cons’ to smoking or quitting could potentially help the advisor understand individual’s views on the subject and target interaction accordingly. More research is needed to gain better insight on how these BCTs can be best incorporated into effective interventions. 

## 5. Conclusions

Since 2009 relatively few behavioral interventions tested have proven effective, highlighting the need for improvement in light of the fact that existing behavioral support interventions struggle to make a meaningful impact on pregnant women’s smoking behavior.

From an expert perspective, the fact that smoking is a social norm and quitting is low on women’s list of priorities are the key barriers to quitting smoking in pregnancy, and these were perceived as most influential but also most difficult to address. Women’s desire to protect their baby from harms of smoke was perceived to be an important facilitator to smoking cessation in pregnancy.

Developing new and tailoring existing BCTs to address ‘Social Influences’, ‘Intentions’, ‘Environmental Context’, ‘Knowledge’, ‘Beliefs about Capabilities’ and ‘Emotions’ TDF domains and incorporating these BCTs into behavioral interventions for stopping smoking in pregnancy could potentially enhance the effectiveness of future interventions.

## 6. Directions for Future Research

The data from this study forms basis for next steps of our research, currently being conducted. We are using the Delphi technique [45], to further prioritize the areas that should be addressed in future development of interventions, by collecting consensus from larger groups of experts. Future research should also be concerned with tailoring support to meet specific needs of pregnant women, by developing new or tailoring existing potentially-effective BCTs, which (1) will be pregnancy specific; (2) will address the key B&Fs to smoking cessation in pregnancy and (3) will be relevant and acceptable to the women and the cessation practitioners. This could be achieved by further expert group meetings, Delphi questionnaires and interviews with pregnant smokers. These BCTs should then be trialed to establish which BCTs or BCT combinations are most effective in helping women quit smoking during pregnancy.

## Figures and Tables

**Table 1:** New barriers (B) and facilitators (F) to smoking cessation in pregnancy identified in the expert group meeting.

Classification	Barrier or Facilitator	Classification
‘Individual’ B&Fs—i.e., with potential to be influenced by advisors during support sessions	Women’s lack of understanding of how to correctly use NRT	B
	Women’s lack of understanding of issues of safety around using Nicotine Replacement Therapy (NRT) in pregnancy	B
	Women underestimate their level of addiction	B
	Accurate assessment of the level of tobacco dependence is needed for more appropriate provision of Nicotine Replacement Therapy (NRT) and/or e-cigs	F
	Women don’t necessarily see quitting smoking as a priority in their complex lives	B
	Previous experience of quitting can affect current motivation to quit	B or F
	Having both internal (e.g., for own or baby’s health) and external motivation to quit (e.g., for approval of family)	F
	Women lack self-belief in their ability to stop smoking and stay stopped	B
	Meaningful, consistent and personal information about cessation intervention can improve women’s engagement	F
	Non-existent, inconsistent and conflicting messages from all health professionals/care providers	B
‘Environmental’ B&Fs—i.e., requiring organizational action	Smoking cessation services may not be structured appropriately, inflexible and/or inaccessible to women and significant others	B
	Lack of identified behavior change programs suitable for pregnant smokers based on level of engagement/motivation	B
	Addressing smoking is not sufficiently high on the agenda health professionals/institutions	B
	Lack of follow-up referral systems	B

**Table 2:** Barriers and facilitators to smoking cessation in pregnancy ranked in order of influence on women’s smoking behavior and difficulty to address in behavioral support.

Barriers and Facilitators to Smoking Cessation Experienced by Pregnant Smokers	Rank in Order of Influence on Women’s Smoking Behavior (1 = Greatest Influence)	Rank of Those Ranked as Most Influential in Order of Difficulty to Address by Advisors in Support Sessions (1 = Most Difficult)
Smoking is a social norm, an acceptable behavior in the women’s close social network. ^1^	1	3
Women don’t necessarily see quitting smoking as a priority in their complex lives. ^2^	2	2
Women want to protect their unborn baby from the harm of smoking. ^1^	3	10
Meaningful, consistent and personal information about cessation intervention can improve women’s engagement. ^2^	4	7
Non-existent, inconsistent and conflicting messages from all health professionals/care providers. ^2^	5	5
Women lack self-belief in their ability to stop smoking and stay stopped.^2^	6	6
Smoking is integral to women’s lives and culture. ^1^	7	1
Having both internal (e.g., for own or baby’s health) and external motivations to quit (e.g., for approval of family). ^2^	8	4
Women underestimate the risks or don’t believe they apply to them. ^1^	9	8
Accurate assessment of the level of tobacco dependence is needed for more appropriate provision of NRT and/or e-cigs. ^2^	10	9

^1^ B&Fs derived from systematic review [17]; ^2^ B&Fs identified in the expert group.

**Table 3:** Frequency of behavior change techniques (BCTs) and competencies identified in two or more effective interventions.

Grouping and BCT/Competency	Present in Number of Effective Trials; *n* (%) Total *N* = 14
**BCTs**	
**Goals and Planning**	**11 (79)**
Problem solving ^b^	8 (57)
Goal setting ^b^	7 (50)
Action planning ^b^	4 (29)
Commitment ^a^	2 (14)
**Feedback and monitoring**	**12 (86)**
Feedback on behavior ^a^	3 (21)
Biofeedback ^b^	12 (86)
**Social support**	**7 (50)**
Social support (unspecified) ^b^	7 (50)
**Natural consequences**	**14 (100)**
Information about health consequences ^b^	14 (100)
**Comparison of behavior**	**2 (14)**
Social comparison ^a^	2 (14)
**Repetition and substitution**	**2 (14)**
Graded tasks ^a^	2 (14)
**Comparison of outcomes**	**10 (71)**
Credible expert ^a^	9 (64)
Pros and cons ^a^	4 (29)
**Reward and threat**	**3 (21)**
Reward (outcome) ^b^	3 (21)
**Regulation**	**4 (29)**
Pharmacological support ^a^	2 (14)
Reduce negative emotions ^a^	4 (29)
**Self-belief**	**3 (21)**
Verbal persuasion about capability ^a^	3 (21)
**Competencies**	
**General aspects of the interaction focusing on gathering information**	**13 (93)**
Assess current and past smoking behaviors ^b^	12 (86)
Assess current readiness and ability to quit ^b^	7 (50)
Assess past history of quit attempts ^a^	5 (36)
Assess nicotine dependence ^a^	3 (21)
Assess number of contacts who smoke ^a^	3 (21)
Assess attitudes to smoking ^a^	2 (14)
**General aspects of the interaction focusing on delivering the intervention**	**4 (29)**
Tailor interactions appropriately ^a^	4 (29)

^a^ New BCTs/competencies identified in the current work; ^b^ BCTs/competencies identified both in the current work and previously [18].

**Table 4:** Theoretical Domains Framework (TDF) [27] domains within which barriers and facilitators to smoking cessation in pregnancy lie, and corresponding potentially-effective behavior change techniques (BCTs).

Domain (Definition; [27])	Barriers (B) and Facilitators (F) That Can Be Addressed with the Woman in Behavioral Support	Potentially-Effective BCTs
**Knowledge** (An awareness of the existence of something)	**Women underestimate the risks of smoking in pregnancy or don’t believe they apply to them (B) ^a,c^** **Meaningful, consistent and personal information about cessation intervention can improve women’s engagement (F) ^b,c^** Women’s lack of understanding of how to correctly use Nicotine Replacement Therapy (NRT) (B) ^b^ Understanding that it is desirable to quit smoking in pregnancy (F) ^a^ Poor understanding of risks related to smoking in pregnancy (B) ^a^ Belief that the stress of quitting will be worse for the baby than continuing to smoke (B) ^a^ Women’s lack of understanding of issues of safety around using NRT in pregnancy (B) ^b^	2.2 Feedback on behavior 2.6 Biofeedback (CO monitoring) 5.1 Information about health consequences
**Social Role and Identity** (A coherent set of behaviors and personal qualities displayed in a social setting)	Being a smoking mother is seen as a negative thing (e.g., ‘good mothers’ don’t smoke) (F) ^a^	-
**Beliefs about Capabilities** (Acceptance of the truth, reality or validity about an ability, talent or facility that a person can put to constructive use)	**Women lack self-belief in their ability to stop smoking and stay stopped (B) ^a,c^** Previous experience of quitting can affect current motivation to quit (B/F) ^a^	15.1 Verbal persuasion about capability
**Optimism** (The confidence that things will happen for the best)	Women underestimate their level of addiction (B) ^a^	15.1 Verbal persuasion about capability
**Intentions** (A conscious decision to perform a behavior or a resolve to act in a certain way)	**Women don’t necessarily see quitting smoking as a priority in their complex lives (B) ^b,c^** **Having both internal (e.g., for own or baby’s health) and external motivation to quit (e.g., for approval of family) (F) ^b,c^** **Women want to protect their unborn baby from the harm of smoking (F) ^a,c^** Women want to bring up children in smoke-free environment (F) ^a^	1.2 Problem solving 1.3 Goal setting 1.4 Action planning 1.9 Commitment
**Environmental Context and Resources** (Any circumstances of a person’s situation or environment that discourages or encourages the development of skills and abilities, independence, social competence and adaptive behavior)	**Accurate assessment of the level of tobacco dependence is needed for more appropriate provision of NRT and/or e-cigs (B) ^b,c^** **Non-existent, inconsistent and conflicting messages from all Health professionals/care providers. (B) ^b,c^**	11.1 Pharmacological support
**Social Influences** (Those interpersonal processes that can cause individuals to change their thoughts, feelings or behavior)	**Smoking is integral to women’s lives and culture. (B) ^a,c^** **Smoking is a social norm, an acceptable behavior in the women’s close social network. (B) ^a,c^** Quitting is just for pregnancy; women and their social circle expect that she will go back to smoking after birth. (B) ^a^ Feeling that others disapprove of smoking in pregnancy can make women hide their smoking. (B) ^a^ Feeling that others disapprove of smoking in pregnancy can lead to quitting smoking. (F) ^a^ Partners’ continued smoking. (B) ^a^Supportive partners (F) ^a^ Lack of support from partners to quit. (B) ^a^ Lack of support from family to quit. (B) ^a^ Support and encouragement from family (F) ^a^ Quitting can make women feel left out if their partner/friends continue to smoke. (B) ^a^ Positive relationships with health professional based on trust and mutual respect. (F) ^a^	3.1 Social support unspecified 6.1 Social comparison 9.1 Credible source
**Emotion** (A complex reaction pattern involving experiential, behavioral and physiological elements by which the individual attempts to deal with a personally significant matter or event)	Smoking can help women cope with everyday stress. (B) ^a^ Fragile mental well-being could be made worse by attempting to stop. (B) ^a^ Fear that quitting smoking could lead to excessive weight gain. (B) ^a^ Sense of guilt could facilitate attempts to quit smoking. (B) ^a^ Smoking gives women pleasure or brief time out (B) ^a^ Smoking can help ease boredom (B) ^a^	11.2 Reduce negative emotions

^a^ B&Fs derived from the systematic review [17]; ^b^ B&Fs identified by the experts in the expert group meeting; ^c^ B&Fs ranked as having the greatest influence on women’s smoking behavior by the experts in the expert group meeting (emboldened).

## Appendix A

List A1. The combined behavior change techniques taxonomy

Behavior Change Techniques Taxonomy v1 (BCTTv1) [16]

1. Goals and planning
1.1. Goal setting (behavior)
1.2. Problem solving (b)
1.3. Goal setting (outcome) (b)
1.4. Action planning (b)
1.5. Review behavior goal(s)
1.6. Discrepancy between current behavior and goal
1.7. Review outcome goal(s)
1.8. Behavioral contract
1.9. Commitment (a)
2. Feedback and monitoring
2.1. Monitoring of behavior by others without feedback
2.2. Feedback on behavior (a)
2.3. Self-monitoring of behavior
2.4. Self-monitoring of outcome(s) of behavior
2.5. Monitoring of outcome(s) of behavior without feedback
2.6. Biofeedback (b)
2.7. Feedback on outcome(s) of behavior
3. Social support
3.1. Social support (unspecified) (b)
3.2. Social support (practical)
3.3. Social support (emotional)
4. Shaping knowledge
4.1. Instruction on how to perform the behavior
4.2.Information about antecedents
4.3.Re-attribution
4.4.Behavioral experiments
5. Natural consequences
5.1. Information about health consequences (b)
5.2. Salience of consequences
5.3. Information about social and environmental consequences
5.4. Monitoring of emotional consequences
5.5. Anticipated regret
5.6. Information about emotional consequences
6. Comparison of behavior
6.1. Demonstration of the behavior
6.2. Social comparison (a)
6.3. Information about others’ approval
7. Associations
7.1. Prompts/cues
7.2. Cue signaling reward
7.3. Reduce prompts/cues
7.4. Remove access to the reward
7.5. Remove aversive stimulus
7.6. Satiation
7.7. Exposure
7.8. Associative learning
8. Repetition and substitution
8.1. Behavioral practice/rehearsal
8.2. Behavior substitution
8.3. Habit formation
8.4. Habit reversal
8.5. Overcorrection
8.6. Generalization of target behavior
8.7. Graded tasks (a)
9. Comparison of outcomes
9.1. Credible source (a)
9.2. Pros and cons (a)
9.3. Comparative imagining of future outcomes
10. Reward and threat
10.1. Material incentive (behavior)
10.2. Material reward (behavior)
10.3. Non-specific reward
10.4. Social reward
10.5. Social incentive
10.6. Non-specific incentive
10.7. Self-incentive
10.8. Incentive (outcome)
10.9. Self-reward
10.10. Reward (outcome) (b)
10.11. Future punishment
11. Regulation
11.1. Pharmacological support (a)
11.2. Reduce negative emotions (a)
11.3. Conserving mental resources
11.4. Paradoxical instructions
12. Antecedents
12.1. Restructuring the physical environment
12.2. Restructuring the social environment
12.3. Avoidance/reducing exposure to cues for the behavior
12.4. Distraction
12.5. Adding objects to the environment
12.6. Body changes
13. Identity
13.1. Identification of self as role model
13.2. Framing/reframing
13.3. Incompatible beliefs
13.4. Valued self-identify
13.5. Identity associated with changed behavior
14. Scheduled consequences
14.1. Behavior cost
14.2. Punishment
14.3. Remove reward
14.4. Reward approximation
14.5. Rewarding completion
14.6. Situation-specific reward
14.7. Reward incompatible behavior
14.8. Reward alternative behavior
14.9. Reduce reward frequency
14.10. Remove punishment
15. Self-belief
15.1. Verbal persuasion about capability (a)
15.2. Mental rehearsal of successful performance
15.3. Focus on past success
15.4. Self-talk
16. Covert learning
16.1. Imaginary punishment
16.2. Imaginary reward
16.3. Vicarious consequences

Smoking-specific competencies [19], which did not map onto the BCTTv1

General aspects of the interaction (R) focusing on the delivery of the intervention (D)
RD1 Tailor interactions appropriately (a)
RD2 Emphasize choice
General aspects of the interaction (R) focusing on information gathering (I)
RI1 Assess current and past smoking behavior (b)
RI2 Assess current readiness and ability to quit (b)
RI3 Assess past history of quit attempts (a)
RI4 Assess withdrawal symptoms
RI5 Assess nicotine dependence (a)
RI6 Assess number of contacts who smoke (a)
RI7 Assess attitudes to smoking (a)
RI8 Assess level of social support
RI9 Explain how tobacco dependence develops
RI10 Assess physiological and mental functioning
General aspects of the interaction (R) focusing on general communication (C)
RC1 Build general rapport
RC2 Elicit and answer questions
RC3 Explain the purpose of carbon monoxide monitoring
RC4 Explain expectations regarding treatment program
RC7 Use reflective listening
RC8 Elicit client views
RC9 Summarize information/confirm client decisions
RC10 Provide reassurance
  (a) New BCTs/competencies identified in the current work
  (b) BCTs/competencies identified both in the current work and previously [18]

## References

[1] Royal College of Physicians (1992). Smoking and the Young. A Report of a Working Party of the Royal College of Physicians.

[2] Batstra L., Hadders-Algra M., Neeleman J. (2003). Effect of antenatal exposure to maternal smoking on behavioural problems and academic achievement in childhood: Prospective evidence from a Dutch birth cohort. Early Hum. Dev..

[3] Rogers J.M. (2008). Tobacco and pregnancy: Overview of exposures and effects. Birth Defects Res. C Embryo Today.

[4] NHS Digital Statistics on Women’s Smoking Status at Time of Delivery. England: April 2016 to March 2017. https://digital.nhs.uk/catalogue/PUB24222.

[5] Al-Sahab B., Saqib M., Hauser G., Tamim H. (2010). Prevalence of smoking during pregnancy and associated risk factors among Canadian women: A national survey. BMC Pregnancy Childbirth.

[6] Curtin S.C., Matthews T.J. (2016). Smoking prevalence and cessation before and during pregnancy: Data from the birth certificate, 2014. Natl. Vital Stat. Rep..

[7] McAndrew F., Thompson J., Fellows L., Large A., Speed M., Renfrew M.J. (2012). Infant Feeding Survey 2010: Health and Social Care Information Centre. http://digital.nhs.uk/catalogue/PUB08694.

[8] Walsh R.A., Redman S., Brinsmead M.W., Fryer J.L. (1997). Predictors of smoking in pregnancy and attitudes and knowledge of risks of pregnant smokers. Drug Alcohol Rev..

[9] Riaz M., Lewis S., Naughton F., Ussher M. (2017). Predictors of smoking cessation during pregnancy: A systematic review and meta-analysis. Addiction.

[10] Taylor A.E., Howe L.D., Heron J.E., Ware J.J., Hickman M., Munafò M.R. (2014). Maternal smoking during pregnancy and offspring smoking initiation: Assessing the role of intrauterine exposure. Addiction.

[11] World Health Organization (2013). WHO Recommendations for the Prevention and Management of Tobacco Use and Second-Hand Smoke Exposure in Pregnancy.

[12] Smoking in Pregnancy Challenge Group (2015). Smoking Cessation in Pregnancy. A Review of the Challenge. http://www.ash.org.uk/files/documents/ASH_979.pdf.

[13] Chamberlain C., O’Mara-Eves A., Porter J., Coleman T., Perlen S.M., Thomas J., McKenzie J.E. (2017). Psychosocial interventions for supporting women to stop smoking in pregnancy. Cochrane Database Syst. Rev..

[14] Craig P., Dieppe P., Macintyre S., Michie S., Nazareth I., Petticrew M. (2008). Developing and evaluating complex interventions: The new Medical Research Council guidance. BMJ.

[15] Michie S., Atkins L., West R. (2014). The Behaviour Change Wheel: A Guide to Designing Interventions.

[16] Michie S., Richardson M., Johnston M., Abraham C., Francis J., Hardeman W., Eccles M.P., Cane J., Wood C.E. (2013). The behavior change technique taxonomy (v1) of 93 hierarchically clustered techniques: Building an international consensus for the reporting of behavior change interventions. Ann. Behav. Med..

[17] Flemming K., McCaughan D., Angus K., Graham H. (2015). Qualitative systematic review: Barriers and facilitators to smoking cessation experienced by women in pregnancy and following childbirth. J. Adv. Nurs..

[18] Lorencatto F., West R., Michie S. (2012). Specifying evidence-based behavior change techniques to aid smoking cessation in pregnancy. Nicotine Tob. Res..

[19] Michie S., Churchill S., West R. (2011). Identifying evidence-based competences required to deliver behavioural support for smoking cessation. Ann. Behav. Med..

[20] Cane J., O’Connor D., Michie S. (2012). Validation of the theoretical domains framework for use in behaviour change and implementation research. Implement. Sci..

[21] Bussières A.E., Patey A.M., Francis J.J., Sales A.E., Grimshaw J.M. (2012). Identifying factors likely to influence compliance with diagnostic imaging guideline recommendations for spine disorders among chiropractors in North America: A focus group study using the Theoretical Domains Framework. Implement. Sci..

[22] Dyson J., Lawton R., Jackson C., Cheater F. (2011). Does the use of a theoretical approach tell us more about hand hygiene behaviour? The barriers and levers to hand hygiene. J. Infect. Prev..

[23] Fulton E.A., Brown K.E., Kwah K.L., Wild S. (2016). StopApp: Using the behaviour change wheel to develop an app to increase uptake and attendance at NHS Stop Smoking Services. Healthcare.

[24] Murphy M.K., Black N.A., Lamping D.L., McKee C.M., Sanderson C.F., Askham J., Marteau T. (1998). Consensus development methods, and their use in clinical guideline development. Health Technol. Assess..

[25] Jones J., Hunter D. (1995). Consensus methods for medical and health services research. BMJ.

[26] McHugh M.L. (2012). Interrater reliability: The kappa statistic. Biochem. Med..

[27] Atkins L., Francis J., Islam R., O’Connor D., Patey A., Ivers N., Foy R., Duncan E.M., Colquhoun H., Grimshaw J.M. (2017). A guide to using the Theoretical Domains Framework of behaviour change to investigate implementation problems. Implement. Sci..

[28] Cane J., Richardson M., Johnston M., Ladha R., Michie S. (2015). From lists of behaviour change techniques (BCTs) to structured hierarchies: Comparison of two methods of developing a hierarchy of BCTs. Br. J. Health Psychol..

[29] Michie S., Johnston M., Francis J., Hardeman W., Eccles M. (2008). From theory to intervention: Mapping theoretically derived behavioural determinants to behaviour change techniques. Appl. Psychol. Int. Rev..

[30] Burling T.A., Bigelow G.E., Robinson J., Mead A.M. (1991). Smoking during pregnancy: Reduction via objective assessment and directive advice. Behav. Ther..

[31] Donatelle R.J., Prows S.L., Champeau D., Hudson D. (2000). Randomised controlled trial using social support and financial incentives for high risk pregnant smokers: Significant other supporter (SOS) program. Tob. Control.

[32] Dornelas E.A., Magnavita J., Beazoglou T., Fischer E.H., Oncken C., Lando H., Greene J., Barbagallo J., Stepnowski R., Gregonis E. (2006). Efficacy and cost-effectiveness of a clinic-based counseling intervention tested in an ethnically diverse sample of pregnant smokers. Patient Educ. Couns..

[33] Ershoff D.H., Mullen P.D., Quinn V.P. (1989). A randomized trial of a serialized self-help smoking cessation program for pregnant women in an HMO. Am. J. Public Health.

[34] Hegaard H.K., Kjaergaard H., Møller L.F., Wachmann H., Ottesen B. (2003). Multimodal intervention raises smoking cessation rate during pregnancy. Acta Obstet. Gynecol. Scand..

[35] Hjalmarson A.I., Hahn L., Svanberg B. (1991). Stopping smoking in pregnancy: Effect of a self-help manual in controlled trial. Br. J. Obstet. Gynaecol..

[36] Lawrence T., Aveyard P., Evans O., Cheng K.K. (2003). A cluster randomised controlled trial of smoking cessation in pregnant women comparing interventions based on the transtheoretical (stages of change) model to standard care. Tob. Control.

[37] Ondersma S.J., Svikis D.S., Lam P.K., Connors-Burge V.S., Ledgerwood D.M., Hopper J.A. (2012). A randomized trial of computer- delivered brief intervention and low-intensity contingency management for smoking during pregnancy. Nicotine Tob. Res..

[38] Polanska K., Hanke W., Sobala W., Lowe J.B. (2004). Efficacy and effectiveness of the smoking cessation program for pregnant women. Int. J. Occup. Med. Environ. Health.

[39] Tappin D., Bauld L., Purves D., Boyd K., Sinclair L., MacAskill S., McKell J., Friel B., McConnachie A., de Caestecker L. (2015). Financial incentives for smoking cessation in pregnancy: Randomised controlled trial. BMJ.

[40] Valbos A., Nylander G. (1994). Smoking cessation in pregnancy. Intervention among heavy smokers. Acta Obstet. Gynecol. Scand..

[41] Walsh R.A., Redman S., Brinsmead M.W., Byrne J.M., Melmeth A. (1997). A smoking cessation program at a public antenatal clinic. Am. J. Public Health.

[42] Windsor R.A., Cutter G., Morris J., Reese Y., Manzella B., Bartlett E.E., Samuelson C., Spanos D. (1985). The effectiveness of smoking cessation methods for smokers in public health maternity clinics: A randomized trial. Am. J. Public Health.

[43] Secker-Walker R.H., Solomon L.J., Geller B.M., Flynn B.S., Worden J.K., Skelly J.M., Mead P.B. (1997). Modeling smoking cessation: Exploring the use of a videotape to help pregnant women quit smoking. Women Health.

[44] Lumley J., Chamberlain C., Dowswell T., Oliver S., Oakley L., Watson L. (2009). Interventions for promoting smoking cessation during pregnancy. Cochrane Database Syst. Rev..

[45] Hsu C.-C., Sandford B.A. (2007). The Delphi technique: Making sense of consensus. Pract. Assess. Res. Eval..

[46] Davidson K.W., Goldstein M., Kaplan R.M., Kaufmann P.G., Knatterud G.L., Orleans C.T., Spring B., Trudeau K.J., Whitlock E.P. (2003). Evidence-based behavioral medicine: What is it and how do we achieve it?. Ann. Behav. Med..

[47] Martin J., Chater A., Lorencatto F. (2013). Effective behaviour change techniques in the prevention and management of childhood obesity. Int. J. Obes. (Lond.).

[48] Lorencatto F., West R., Stavri Z., Michie S. (2013). How well is intervention content described in published reports of smoking cessation interventions?. Nicotine Tob. Res..

[49] West R., Walia A., Hyder N., Shahab L., Michie S. (2010). Behavior change techniques used by the English Stop Smoking Services and their associations with short-term quit outcomes. Nicotine Tob. Res..

[50] Michie S., van Stralen M.M., West R. (2011). The behaviour change wheel: A new method for characterising and designing behaviour change interventions. Implement. Sci..

[51] Nguyen S.N., Von Kohorn I., Schulman-Green D., Colson E.R. (2012). The importance of social networks on smoking: Perspectives of women who quit smoking during pregnancy. Matern. Child Health J..

[52] Bauld L., Graham H., Sinclair L., Flemming K., Naughton F., Ford A., McKell J., McCaughan D., Hopewell S., Angus K. (2017). Barriers to and facilitators of smoking cessation in pregnancy and following childbirth: Literature review and qualitative study. Health Technol. Assess..

[53] Ingall G., Cropley M. (2010). Exploring the barriers of quitting smoking during pregnancy: A systematic review of qualitative studies. Women Birth.

[54] Sloan M., Campbell K.A., Bowker K., Coleman T., Cooper S., Brafman-Price B., Naughton F. (2016). Pregnant women’s experiences and views on an “opt-out” referral rathway to specialist smoking cessation support: A qualitative evaluation. Nicotine Tob. Res..

[55] Abatemarco D.J., Steinberg M.B., Delnevo C.D. (2007). Midwives’ knowledge, perceptions, beliefs, and practice supports regarding tobacco dependence treatment. J. Midwifery Womens Health.

[56] Campbell K., Bowker K., Naughton F., Sloan M., Cooper S., Coleman T. (2016). Antenatal clinic and stop smoking services staff siews on “opt-out” referrals for smoking cessation in pregnancy: A framework analysis. Int. J. Environ. Res. Public Health.

[57] Bowker K., Campbell K.A., Coleman T., Lewis S., Naughton F., Cooper S. (2015). Understanding pregnant smokers’ adherence to nicotine replacement therapy during a quit attempt: A qualitative study. Nicotine Tob. Res..

[58] Glover M., Kira A. (2012). Pregnant Māori smokers’ perception of cessation support and how it can be more helpful. J. Smok. Cessat..

[59] England L.J., Tong V.T., Koblitz A., Kish-Doto J., Lynch M.M., Southwell B.G. (2016). Perceptions of emerging tobacco products and nicotine replacement therapy among pregnant women and women planning a pregnancy. Prev. Med. Rep..

[60] Raupach T., Brown J., Herbec A., Brose L., West R. (2014). A systematic review of studies assessing the association between adherence to smoking cessation medication and treatment success. Addiction.

[61] Bowker K., Lewis S., Coleman T., Cooper S. (2015). Changes in the rate of nicotine metabolism across pregnancy: A longitudinal study. Addiction.

[62] Gamble J., Grant J., Tsourtos G. (2015). Missed opportunities: A qualitative exploration of the experiences of smoking cessation interventions among socially disadvantaged pregnant women. Women Birth.

[63] Taylor G., McNeill A., Girling A., Farley A., Lindson-Hawley N., Aveyard P. (2014). Change in mental health after smoking cessation: Systematic review and meta-analysis. BMJ.

[64] Ripley-Moffitt C.E., Goldstein A.O., Fang W.L., Butzen A.Y., Walker S., Lohr J.A. (2008). Safe babies: A qualitative analysis of the determinants of postpartum smoke-free and relapse states. Nicotine Tob. Res..

[65] Campbell K.A., Cooper S., Fahy S.J., Bowker K., Leonardi-Bee J., McEwen A., Whitemore R., Coleman T. (2017). ‘Opt-out’ referrals after identifying pregnant smokers using exhaled air carbon monoxide: Impact on engagement with smoking cessation support. Tob. Control.

[66] Lawrence T., Aveyard P., Croghan E. (2003). What happens to women’s self-reported cigarette consumption and urinary cotinine levels in pregnancy?. Addiction.

[67] National Institute for Health and Clinical Excellence (2010). Public Health Guidance 26: How to Stop Smoking in Pregnancy and Following Childbirth; PH26. http://guidance.nice.org.uk/PH26/Guidance/pdf/English.

[68] Murphy D.J. (2015). Financial rewards for pregnant smokers who quit. Br. Med. J..

[69] McCartney M. (2015). The ethics of behavioural incentives. Br. Med. J..

